# Preachers, Patent Medicines and Doctors’ Bills

**Published:** 1891-01

**Authors:** 


### PREACHERS, PATENT MEDICINES AND DOCTORS' BILLS.

---

Our esteemed contemporary, the *Dixie Doctor*, has, in its last two issues, been making a vigorous and incisive warfare against those ministers of the gospel who, from mercenary motives, lend themselves to the quack and patent medicine vendor. Too strong language cannot be applied to such impostors. But it does not necessarily follow that every preacher who gives a certificate extolling the virtues of some patent medicine which he may have conceived to have benefited him is a knave. Preachers are not educated in medical lore—some not at all—and many who are thoroughly educated from a purely literary standpoint have sorely neglected the scientific sides of themselves, and hence fall a ready prey to the slick-tongued agent who has furnished them with a cure-all for family use. Some may honestly believe that they are doing the good people a great service by certifying to a favorite nostrum. But does ignorance condone the offense ? Why should a preacher possess peculiar powers of discrimination as to the virtues of patent medicines ? Has that been a part of his education ? Then why assume knowledge which he does not possess ? They cannot have thought of the harm they have been doing. They are not aware, as we are, of the baleful effects of patent medicine tippling. They do not know the evil effects of self-diagnosing and constant self-prescribing. They are not brought face to face with the deplorable wrecks who have ruined mind and body by unceasing dread of disease and everlasting dosing for imaginary ills. They do not know that most of the vaunted nostrums owe to the alcohol which they contain whatever of virtue they may possess, and that they call for constantly increasing doses, and not infrequently lead to alcoholic intemperance. We congratulate our contemporary upon the good fight it is making against this prevailing evil. But we must demur from the sweeping condemnation of preachers for not paying physicians for professional services. They are arraigned for that which we should lay at our own doors. They do not pay us fees simply because we will not accept them. Most of them would no doubt prefer to pay their doctors just as they do their grocers. Of course they do not expect to pay physicians, because the custom of not charging ministers is almost universal. Whether it be a good custom or not, the preachers are not to be censured for it. If the custom is a bad one, we are responsible for its continuance, as were our predecessors for its origin.

